# Editorial: Sequelae of Prostate Cancer Therapy: Avoidance Strategies and Management Options

**DOI:** 10.3389/fsurg.2022.849669

**Published:** 2022-04-06

**Authors:** Clemens M. Rosenbaum, Felix Campos-Juanatey, Luis A. Kluth, for the Trauma Reconstructive Urology Working Party of the European Association of Urology Young Academic

**Affiliations:** ^1^Department of Urology, Asklepios Hospital Barmbek, Hamburg, Germany; ^2^Department of Urology, Marqués de Valdecilla University Hospital, Santander, Spain; ^3^Department of Urology, University Hospital Frankfurt, Goethe University Frankfurt, Frankfurt, Germany

**Keywords:** urinary incontinence, prostate neoplasia, erectile dysfunction, urethral stricture, radiation, prostatectomy, focal therapy

Prostate cancer is the most common malignancy among men in the Western world (1). More than 80% of patients with clinically localized prostate cancer will undergo definite treatment (2). Most common treatment options are radical prostatectomy and radiotherapy, focal therapies such as high-intensity focused ultrasound (HIFU) or cryoablation being increasingly used. All of them come along with different patterns of early and late side effects (3). Given excellent survival rates at 10 years (4), urologists have to face a relevant number of patients who present with one of these prostate cancer treatment related sequelae.

The goal of our Research Topic “Sequelae of Prostate Cancer Therapy: Avoidance Strategies and Management Options” was therefore to provide readers, researchers and physicians a comprehensive overview of strategies to prevent consequences of prostate cancer therapies and future perspectives of management of sequelae of prostate cancer treatments.

One of the most common side effects of prostate cancer treatment is urinary incontinence (5). Prostatectomy has worse effects on urinary incontinence compared to radiation therapy (5). Rahnama'i et al. illustrate the current knowledge of how to avoid urinary incontinence during radical prostatectomy. Besides surgical factors, patient characteristics as higher body mass index, older age, pre-existing lower urinary tract symptoms, neurological disease and functional bladder changes, have been identified to negatively impact continence (6). Lately, sarcopenia, defined as low skeletal muscle volume, has been increasingly recognized as a potential risk factor for worse outcome in oncologic patients. However, Angerer et al. were able to show that it has no influence on post-prostatectomy continence rates. As treatment of post-prostatectomy caused urinary incontinence, the artificial urinary sphincter has been considered the gold standard for several decades. Rahnama'i et al. demonstrated in their review several alternative surgical procedures that challenge the artificial urinary sphincter (6).

Another common consequence of prostate cancer treatment is erectile dysfunction (5). Sparing neurovascular bundles during surgery is the most important factor to maintain erectile function. Besides nerve-sparing surgery, methods for penile rehabilitation after radical prostatectomy and radiation therapy are focus of current research. Nicolai et al. give an overview about pathophysiology and treatment of erectile dysfunction following radical prostatectomy. In addition, Schoentgen et al. are able to show in their systematic review, that sexual rehabilitation prior to radical prostatectomy may result in better erectile recovery.

For both urinary incontinence and erectile dysfunction, tissue engineering could help to overcome the current borders of treatment. Autologous stem cell transplantation is one of the most promising approaches. Adamowicz et al. describe a tissue engineering approach, mode of vascular and neuro-regeneration and stem cell safety. They are able to illustrate the unquestionable potential of tissue engineering to improve outcome of prostate cancer treatment related sequelae (Adamowicz et al.).

Furthermore, bladder outlet obstruction is a common problem not only after radical prostatectomy but also after radiation therapy.

The review “Contemporary Management of Vesico-Urethral Anastomotic Stenosis After Radical Prostatectomy” gives an overview about pathophysiology and treatment of vesicourethral anastomotic stenosis. The authors demonstrate endourological procedures should still remain as an initial treatment. However, in refractory stenoses, open or robotic reconstruction is a viable option with high success rates (Rosenbaum et al.). In contrast to vesicourethral anastomotic stenosis after radical prostatectomy, radiation induced membranous urethral strictures may occur years after therapy. Waterloos et al. illustrate that management of radiation induced urethral strictures can be challenging. Poor vascularized tissue and the proximity of the sphincter can impair functional outcomes (Waterloos et al.).

Devasted bladder outlet or radiogenic chronic cystitis are rare complications after prostate cancer treatments, but can have a huge impact on quality of life. Hoeh et al. provide an overview about treatment options in these patients, in which urinary diversion may also be discussed as a definite treatment.

Most of the aforementioned problems result of surgery or radiation. Focal therapy aims to selectively treat the part of the prostate that harbors significant prostate cancer while preserving the rest of the gland. Aim of this therapeutic approach is to retain the oncological benefit of active treatment while minimizing side-effects. Most common complications of focal therapy are urinary tract infections, acute urinary retention, dysuria and haematuria, however, urinary incontinence is rare. In the salvage setting, after external beam radiation therapy, focal therapy has a significantly higher rate of severe complications. Rakauskas et al. give a comprehensive overview.

Finally, all type of treatment inherit the risk of recurrence. After radical prostatectomy, the role and timing of radiation therapy remains highly controversial (7–9). Zattoni et al. give a comprehensive overview about the currently ongoing discussion. Still, about 40% of patients develop biochemical recurrence within 10 years after primary therapy (10). Limited sensitivity and specificity of conventional imaging methods, such as computed tomography and magnetic resonance imaging has led to efforts in developing better modalities. Lately PSMA-PET/CT has been introduced as such. Initially promising results have been confirmed. Leitsmann et al. are able to demonstrate that mesorectal lymph node metastases detected by PSMA-PET/CT seem to be a relevant localization of tumor recurrence after active therapy. They may serve as index lesion in the treatment of recurrent prostate cancer.

Prostate Cancer remains one of the major parts of Urology. Primary treatment of prostate cancer and management of recurrences is one side of the coin, while the other side is dealing treatment of sequalae of initial or recurrent treatment.

## Author Contributions

CR, FC-J, and LK: manuscript writing. All authors contributed to the article and approved the submitted version.

## Conflict of Interest

The authors declare that the research was conducted in the absence of any commercial or financial relationships that could be construed as a potential conflict of interest.

## Publisher's Note

All claims expressed in this article are solely those of the authors and do not necessarily represent those of their affiliated organizations, or those of the publisher, the editors and the reviewers. Any product that may be evaluated in this article, or claim that may be made by its manufacturer, is not guaranteed or endorsed by the publisher.
